# Use of big data from health insurance for assessment of cardiovascular outcomes

**DOI:** 10.3389/frai.2023.1155404

**Published:** 2023-05-03

**Authors:** Johannes Krefting, Partho Sen, Diana David-Rus, Ulrich Güldener, Johann S. Hawe, Salvatore Cassese, Moritz von Scheidt, Heribert Schunkert

**Affiliations:** ^1^Department of Cardiology, Deutsches Herzzentrum München, Technische Universität München, Munich, Germany; ^2^German Center for Cardiovascular Research e.V. (DZHK), Partner Site Munich Heart Alliance, Munich, Germany

**Keywords:** machine learning, healthcare research, health insurance claims, prediction, artificial intelligence, big data, prevention

## Abstract

Outcome research that supports guideline recommendations for primary and secondary preventions largely depends on the data obtained from clinical trials or selected hospital populations. The exponentially growing amount of real-world medical data could enable fundamental improvements in cardiovascular disease (CVD) prediction, prevention, and care. In this review we summarize how data from health insurance claims (HIC) may improve our understanding of current health provision and identify challenges of patient care by implementing the perspective of patients (providing data and contributing to society), physicians (identifying at-risk patients, optimizing diagnosis and therapy), health insurers (preventive education and economic aspects), and policy makers (data-driven legislation). HIC data has the potential to inform relevant aspects of the healthcare systems. Although HIC data inherit limitations, large sample sizes and long-term follow-up provides enormous predictive power. Herein, we highlight the benefits and limitations of HIC data and provide examples from the cardiovascular field, i.e. how HIC data is supporting healthcare, focusing on the demographical and epidemiological differences, pharmacotherapy, healthcare utilization, cost-effectiveness and outcomes of different treatments. As an outlook we discuss the potential of using HIC-based big data and modern artificial intelligence (AI) algorithms to guide patient education and care, which could lead to the development of a learning healthcare system and support a medically relevant legislation in the future.

## Graphical Abstract

Amount of data from individual treatment units. The volume of medical data in healthcare systems globally is continually expanding. While data access to individual patient data is limited over time for individual treatment units, relevant data from all healthcare providers converge at health insurance companies. However, access to insurance data for health services research is still constrained. We illustrate that utilizing this data can significantly enhance risk prediction, prevention, and patient care. 
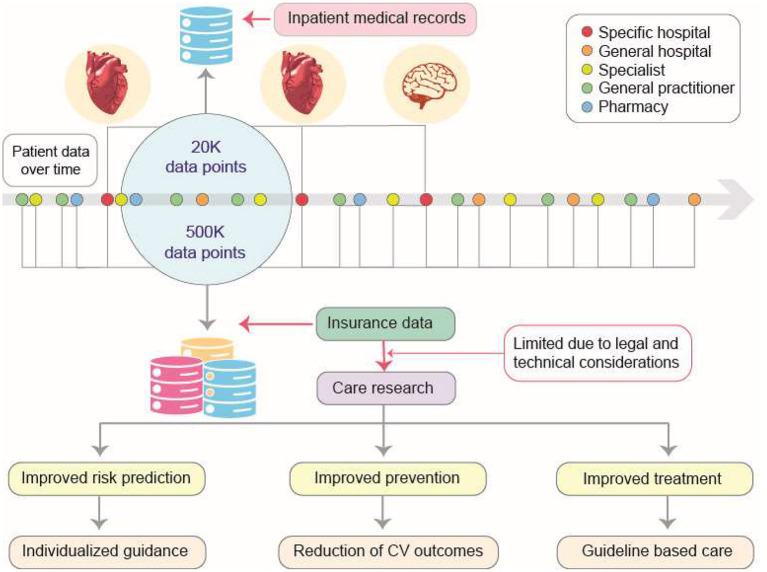

## Introduction

The volume of data generated by healthcare systems has been growing exponentially over the past decade. Introduction of electronic medical records and establishment of smart devices in medicine will massively drive this development even further. In 2020, the healthcare systems were estimated to globally generate 2.3 zettabytes (1 zettabyte = 1 trillion gigabytes) of data, which will triple by 2025 (Jeffrey, 2022). A big part of medical patient data is available in the form of health insurance claims (HIC). In many countries, HIC data have already been used to infer health status at the population level resulting in new medical findings (Raghupathi and Raghupathi, 2014). While randomized controlled trials (RCTs) are the unchallenged state-of-the-art strategy to obtain highest quality insights for clinical decision making, insurance data can be used to obtain information where RCTs are not feasible. An important example is patient's out-of-hospital life in between healthcare utilizations, for which insurance claims data can aggregate health care utilizations, including ambulatory care, providing more complete insights about the patient's health over the course of time. Moreover, insurance claims data could help to understand patient's utilization of medication and other out-of-hospital health related activities. This information could aid in addressing the impending demographic shifts, such as an aging population and an increase in comorbidities. A better understanding of the health status could lead to a better treatment whilst containing the costs of the health systems. However, the utilization of HIC data to enhance healthcare is limited by various infrastructural and legal barriers, including concerns surrounding data security (Graphical Abstract).

Cardiovascular diseases (CVD) are the main causes of death worldwide, and cause highest financial burden in most healthcare systems (Eurostat Statistics Explained, 2022; Statistisches Bundesamt, 2022; World Health Organization, 2022). HIC data could help to better understand CVD risk factors and inform guidelines to decrease morbidity and mortality. Furthermore, they may identify opportunities of disease prevention or occurrence of secondary events, and consequently improve cost-effectiveness of the healthcare system. Finally, the usage of HIC data can contribute significantly to the quality assessments of cardiac care (Rumsfeld et al., 2016). Our review focuses on the analysis of HIC data to provide information on individuals with CVD and aims to emphasize the use of existing health data to improve global health.

## History, established principles and current state of the art

Since the introduction of the world's first “Bismarckian” statutory health insurance (SHI) in 1883 many countries have introduced mandatory SHI for most citizens. An important step toward the use of the data from respective insurance claims for statistical analysis was the establishment of coding systems. Coding allows for an objective and universal labeling of clinical information about patients. The most important coding tool is “The International Statistical Classification of Diseases and Related Health Problems” (ICD), which was officially introduced as International List of Causes of Death (ILCD) in 1900 at the “First International Conference for the Revision of the Bertillon or International List of Causes of Death” (Hirsch et al., 2016). Since then, it has been continuously developed from a register of causes of death toward a register of diseases, which are not necessarily leading to death but impairing health. Currently, ICD-10 is used by over 100 countries, distinctly defining over 70,000 diseases and their subclasses (ICD Codes, 2022). ICD-11 has already been introduced at the beginning of 2022 and is ought to be adapted by 2026. Other important coding-systems concern the performed procedures and are used for the reimbursement systems of health insurance companies, namely the “Current Procedural Terminology” (CPT) in the United States of America (USA) (Hirsch et al., 2015) or the “Operationen- und Prozedurenschlüssel” (OPS) in Germany (Bundesinstitut für Arzneimittel und Medizinprodukte, 2022). To monitor international drug utilization the Anatomical Therapeutic Chemical (ATC)—Classification in combination with the Defined Daily Dose (DDD) are used (WHO Collaborating Centre for Drug Statistics Methodology, 2022). This provides useful information about the actual pharmaceutical therapy of each patient.

The analysis of medical statistics dates back to 1662 when John Graunt wrote his work about “Natural and Political Observations Made upon the Bills of Mortality” (Federal Institute for Drugs Medical Devices, 2022). The analysis of insurance claims data for clinical findings steadily grew over the last years (Sawicki et al., 2020). Initially descriptive statistical methods were used, recently more advanced statistical and machine learning (ML) approaches have been employed in the clinical research (van der Galiën et al., 2021). Furthermore, there is a massive interest in the integration of multiple databases from different insurance providers (Riedel et al., 2018; Sabaté et al., 2021) or with the clinical data (Jones et al., 2018). Currently there is an ongoing discussion about the establishment of a Health Research Data Center in Germany. The center is meant to be a centralized platform for storing and sharing health-related research data to facilitate research in the field (Swart et al., 2021), to date, however it is still in the process of being established (Bundesinstitut für Arzneimittel und Medizinprodukte, 2023). The potential of large volume of insurance data has already drawn the interest of various institutions and government organizations including the European Union (EU) resulting in European funded initiatives (Pastorino et al., 2019). In the following part, we will highlight the main findings of HIC data analysis in CVD.

## Current findings on cardiovascular diseases from insurance data

The focus of statistical analysis of HIC data can mostly be grouped into five different topics: (a) demographical and epidemiological differences, (b) pharmacotherapy, (c) healthcare utilization, (d) outcomes of different treatments, and (e) cost-effectiveness.

Since HIC data contains information about sex, age, and other demographics; differences in behavior and clinical outcomes of different patient-groups have been evaluated according to these factors. HIC data are well suited for assessing the effectiveness of the actual pharmacotherapy because they capture data on the medications that patients actually obtained from the pharmacy. This information provides a blend of the medication prescribed by physicians and the degree of patient adherence to the treatment. Many studies indicated that there is a discrepancy between the recommended, prescribed pharmaceutical therapy, and what is actually received by the patient (Mangiapane and Busse, 2011; Eindhoven et al., 2018a; Ulrich et al., 2019). Drug adherence is extremely important after myocardial infarction (MI) and stenting to prevent stent thrombosis or a progression of the coronary artery disease (CAD). Sex-differences can be observed regarding adherence to treatment. Analyses of HIC data from the Netherlands and Germany showed that women seem to be less drug adherent to their prescribed medicine after MI (Eindhoven et al., 2018b; Ulrich et al., 2019). Data collected from French HIC showed that males are on average a decade younger than female patients, at the time of hospital-admission for MI (Blin et al., 2016). Regarding the impact of age, HIC data from the Netherland showed that adherence to optimal treatment decreases with an increase in age (Eindhoven et al., 2018b; Beller et al., 2020). Furthermore, the adherence to recommended drugs after MI decreases steadily over time (Ulrich et al., 2019), whilst patients with Non ST-elevation myocardial infarct (NSTEMI) were less frequently treated with the guideline recommended medication to begin with (Eindhoven et al., 2018b). Nevertheless, the overall usage according to the current guidelines is increasing over the last years (Koopman et al., 2013; Blin et al., 2016).

Information can also be gained about prescription and usage patterns of specific medications in pharmacotherapy. Looking at anticoagulation, for example, the overall discontinuation rates of vitamin K antagonists and new oral anticoagulants are comparable over the first year of therapy (Hohnloser et al., 2019), and the highest persistence rate was seen for apixaban (Sabaté et al., 2021). Statins are another drug class with common non-adherence. Here the discontinuation of therapy could be associated with the patients socio-economic status and their utilization of follow-up appointments with physicians (Hickson et al., 2017).

HIC data has been used to evaluate the adaption of new guidelines. For example, Shafazand et al. (2010) highlighted that only small differences in prescribing practices were seen before and after the publication of the ACC/AHA guidelines (2005) for the treatment of heart failure (HF). However, it is important to consider the potential limitations of routine data when interpreting the results of studies that use these data sources to assess guideline concordance. Routine data often lack essential clinical findings, such as laboratory values or physiological parameters like blood pressure or heart frequency, which are important for assessing the quality of care provided to patients.

Every health care utilization is documented in HIC data, if it is reimbursed by the insurer. Therefore, it can be used to analyze the impact of different health care utilizations on the patient's course of disease. German HIC data were used to show that patients visit a general practitioner (GP) or cardiologist at least once a year after MI (Ulrich et al., 2019). Radzimanowski et al. (2017) showed that mortality was three times higher in patients without cardiology care, however sole contact with a cardiologist without visiting a general practitioner (GP) /internist was insufficient to increase the survival rates. However, it also implied that an increased number of visits to the same kind of physician did not account for survival benefit of patients with cardiology care.

HIC data also offers the opportunity to compare the effect of different medical treatments and interventions on certain clinical outcomes and adverse events. Several studies could underline the superiority of drug-eluting stents (DES) and coronary artery bypass grafts (CABG) over bare metal stents (BMS) associated with the occurrence of major adverse cardiac and cerebrovascular events (MACCE) (Jeschke et al., 2017; Nestler et al., 2022). With regard to predicting treatment success in terms of individual risk, Barth et al. (2018) highlighted that recognition of the number of comorbidities in a high-risk patient population undergoing transcatheter aortic valve implantation (TAVI) improved outcome prediction.

It has already been reported before that a small group of patients disproportionately consume a large proportion of healthcare resources (Kim and Park, 2019). HIC data can help to determine the characteristics of these patients, including specific treatments and circumstances that contribute to this significant imbalance in expenses. Furthermore, HIC data demonstrated that cost-effectiveness strongly depends on timing and situation. In patients with heart failure, end-of-life care is significantly more costly compared to the treatment for those who survive their hospital stay (Obi et al., 2017). Among other procedures, percutaneous coronary intervention (PCI) has been shown to be cost-effective across different ages (Liao et al., 2021), with an advantage of DES over BMS (Cheng et al., 2019). In the early days of catheter ablation, the cost-effectiveness of treating atrial fibrillation (AF) was questioned by van Brabandt et al. (2013) who suggested it to be reserved for carefully selected patients.

Regarding peripheral artery disease (PAD) the analysis of German HIC data revealed a significant increase in the diagnosis and associated costs for the German healthcare system (Malyar et al., 2013). However, a subsequent study using the same data found no significant improvement in clinical outcome for PAD patients when compared to historical data. This study attributed the lack of improvement to low rates of follow-up diagnostics and poor adherence to guidelines (Reinecke et al., 2015). Another subsequent study, which also utilized German HIC data, revealed that this was particularly the case for women, despite their superior overall and amputation-free survival rates (Makowski et al., 2022).

Geographic information derived from insurance data can point out disparities in health care utilization at a more granular level, including small-area variations. By analyzing this data, policymakers and healthcare providers can gain a more comprehensive understanding of the impact of health initiatives on specific populations and geographic regions. The analysis of HIC data can highlight the need for targeted investments in healthcare facilities or resources in areas with lower levels of healthcare utilization. Similarly, inspired by the work of Wennberg and others (Wennberg, 2010), it can help to identify regions where overutilization of certain healthcare services may be driving up costs unnecessarily, thereby allowing for more efficient allocation of resources.

## Advantages and challenges of using health insurance claims data

Working with HIC data provides important and copious patient information which cannot easily be obtained using RCTs. While RCTs are often restricted to highly selective cohorts of patients depending on their willingness to give consent or the location of inclusion. Analyzing HIC data offers an overview of all the treated individuals. With millions of patients, HIC data provide a huge study population in which specific subgroups can be studied. In addition, long follow-up periods without missing data due to dropouts are possible if patients remain in the same insurance company. This combination leads to large patient cohorts with long follow-up periods and providing the corresponding study with a high predictive power. Hybrid forms of investigations, utilizing both randomized controlled trial (RCT) methodology and registry data, have been conducted using the SWEDEHEART CR registry as a foundation (Hofmann et al., 2017). These investigations have leveraged the strengths of RCTs, such as the ability to provide a high level of evidence for causal inferences, with the benefits of registry data, including pre-existing infrastructure for patient recruitment and follow-up.

However, HIC data incorporate several limitations. It contains limited in-depth information on individual patients, thereby limiting the number of questions to be addressed. Further, it can be affected by misinterpretation of the given information. However, most information is found in the form of codes regarding the diseases via ICD codes, procedure numbers, and ATCs. For this reason, HIC data are mainly objective. The codes are thoroughly checked by the insurance companies since they provide the basis for reimbursement. However, it is important to critically examine the reliability of these codes. Above all, the assumption of causalities must be made with extreme caution. For instance ICD-Codes may not always accurately reflect the reasons behind drug prescriptions (Gothe, 2008). Furthermore, coding inaccuracy cannot be completely prevented. An accuracy of over 70% for diagnosing acute myocardial infarction (AMI) was shown in Korean HIC data based on ICD-10 codes (Kimm et al., 2012). The diagnoses listed in Medicare even have a positive predictive value of 94% in the USA (Kiyota et al., 2004). Even though the accuracy of coding in most health systems can be accepted as quite accurate, it is essential to carry out validation on provided codes. For certain diagnoses, this can be achieved by combining the ICD codes with the procedural codes. By that way a diagnosis that demands a certain procedure, (for e.g., AMI and stenting or CABG) can be interpreted correctly. An indirect validation could be a change of medication at the occurrence or aggravation of a certain disease.

To ensure that secondary data from HIC are analyzed using rigorous and transparent methods, and to promote accuracy, reliability, and reproducibility of research results, guidelines such as RECORD (Nicholls et al., 2016), the Good Practice Secondary Data Analysis (Swart et al., 2015) or STROSA (Swart et al., 2016) must be followed. These guidelines help researchers identify potential sources of bias and confounding, minimize the impact of these factors on results, and present their methods and results clearly and transparently. They also ensure the secure handling of secondary data in the context of health research.

Depending on the country and type of health insurance, certain insurance populations might not accurately represent the general population of the country regarding social status, sex, and age (Barth et al., 2018; van der Galiën et al., 2021). Therefore, validation studies have been performed for different HIC datasets. For the German population, it was shown that there are differences between STH and privately insured patients, especially with regard to socio-economic status, while gender and age are largely identical (Jaunzeme et al., 2013; Epping et al., 2021). Validation studies from the United Kingdom (UK) and the Netherlands have substantiated the insured population to be representative to their general population and showed sufficient data accuracy (Aylin et al., 2007; Eindhoven et al., 2018a). Jaunzeme et al. (2013) suggested that validity of STH data is not primarily based on their representativeness but on the condition that the relevant characteristics are sufficiently represented in these data in order to carry out a stratification.

Using real world patient data comes with the challenge of data privacy. Since the Health Insurance Portability and Accountability Act of (HIPAA, 1996), limits the use of patients' personal medical information for researchers in the USA (CDC Centers for Disease Control Prevention, 2022). After the enforcement of HIPAA health care providers and other organizations, holding sensible patient data, are obligated to ensure the safety and prevent misuse. This resulted in new necessary provisions such as informed patient consent, which from then on were deemed necessary for the use of patient-related data in research. While that way patient data is protected, it has been shown that a change of the study population due to selection bias is an unwanted side effect (Armstrong et al., 2005). Bypassing informed consent, de-identified patient data are still accessible for research purposes without the need for consent. It has been shown though, that anonymous data can lead to research errors and a decreased amplitude of studies (Wilson, 2006). The General Data Protection Regulation (GDPR) was implemented in the EU and UK in 2018. This privacy law mandates that all organizations within the EU and UK adhere to fundamental data protection principles and safeguard individuals' privacy rights. While HIPAA still permits some degree of patient health information disclosure, the EU law is even more restrictive. Although the protection of patient data is necessary, current data protection laws lead to an increase in the cost and time required to comply with and conduct studies, as well as a loss of information that could be used for the benefit of the population (Wolf and Bennett, 2006).

## Future directions

Most findings from HIC data are based on descriptive statistics, and there is an urgent need to develop, extend and implement software tools and frameworks to analyze the vast quantities of data available. These tools should be able to address multiple questions providing deep insights from the data as compared to the traditional statistical methods such as simple linear regression, which based on linear relationships. Modern AI models can be used to discover more complex relationships. Moreover, HIC data should be used to develop predictive ML-models suggesting health status of the patients. Only a few supervised and unsupervised ML-based approaches have been applied to insurance data (Davenport and Kalakota, 2019; Thesmar et al., 2019; Kaushik et al., 2022), these techniques include decision-tree learning and natural language processing (NLP). However, other ML-based techniques are scarcely applied (Rumsfeld et al., 2016). Using the state of art ML techniques on clinical data combined with HIC data might provide a deeper understanding of the patient's behavior, and improve risk stratification (van der Galiën et al., 2021). Despite the potential benefits of using ML models, caution needs to be applied; training datasets must be adequately defined, unbiased for sex, ethnicity, social and cultural biases. It is crucial to apply ML-based techniques that can accurately predict outcomes in a diverse population (Zou and Schiebinger, 2018). Furthermore, the representativeness of the sample population and potential systematic biases that could impact the model predictions should be made transparent (Ibrahim et al., 2020).

Even though the potential of HIC data is enormous, the findings from the HIC-data have been hardly considered in clinical guidelines and/or clinical practice (Rumsfeld et al., 2016). The coordination between GPs, specialists and hospital doctors could be improved by providing access to all relevant data, including patient's behavior outside the hospital, knowledge of risk factors, a better understanding of needs and specific care requirements. This would enhance patient care outcomes and facilitate monitoring and tracking of patient progress (Eindhoven et al., 2018a).

## Conclusion

Taken together, HIC data are stable and valid data that have been used for the gain of clinical information for decades. Nevertheless, the utilization of HIC data needs to be aligned with appropriate methods and can be improved in terms of analysis and implementation of their findings in clinical practices. An important challenge while using patient data is protection of privacy, which in its current form leads to loss of critical information that might help to complete the clinical picture of patients in hospital and therefore, provide important insights for the treatment of the general population. Using real world big data could create a learning healthcare system, improving cost-effectiveness and patients' outcome. A healthcare system has the potential to provide relevant information, supporting prediction accuracy, decision and policy making. Data driven health policy might aid to educate individuals and prevent diseases. Furthermore, it would facilitate to unburden the increasingly scarce health care resources.

## Author contributions

JK, MS, PS, and DD-R contributed to conception and design of the review. JK wrote the first draft of the manuscript. MS and PS wrote sections of the manuscript. HS and MS supervised the project and writing. All authors contributed to manuscript revision, read, and approved the submitted version.

## References

[B1] Armstrong D. Kline-Rogers E. Jani S. M. Goldman E. B Fang J. Mukherjee D.. (2005). Potential impact of the HIPAA privacy rule on data collection in a registry of patients with acute coronary syndrome. Arch. Intern. Med. 165, 1125–1129. 10.1001/archinte.165.10.1125 15911725

[B2] Aylin P. Bottle A. Majeed A. (2007). Use of administrative data or clinical databases as predictors of risk of death in hospital: comparison of models. BMJ. 334, 1044. 10.1136/bmj.39168.496366.55 17452389 PMC1871739

[B3] Barth A. Yücel S. Ince H. Doblhammer G. (2018). Impact of transcatheter aortic valve implantation on the risk of mortality in patients with severe aortic valve diseases: a health insurance-based analysis. Open Heart. 5, e000756. 10.1136/openhrt-2017-000756 29713484 PMC5922570

[B4] Beller J. Bauersachs J. Schäfer A. Schwettmann L. Heier M. Peters A.. (2020). Diverging trends in age at first myocardial infarction: evidence from two german population-based studies. Sci. Rep. 10, 9610. 10.1038/s41598-020-66291-4 32541657 PMC7296035

[B5] Blin P. Philippe F. Bouée S. Laurendeau C. Torreton E. Gourmelin J.. (2016). Outcomes following acute hospitalised myocardial infarction in France: An insurance claims database analysis. Int. J. Cardiol. (2016). 219: 387–393. 10.1016/j.ijcard.06102 27372604

[B6] Bundesinstitut für Arzneimittel und Medizinprodukte (2022). Operationen- und Prozedurenschlüssel (OPS). Available online at: https://www.bfarm.de/DE/Kodiersysteme/Klassifikationen/OPS-ICHI/OPS/_node.html (accessed December 12, 2022).

[B7] Bundesinstitut für Arzneimittel und Medizinprodukte (2023). Das Forschungsdatenzentrum Gesundheit (FDZ Gesundheit): Forschung für eine optimale Gesundheitsversorgung). Available online at: https://www.forschungsdatenzentrum-gesundheit.de/ (accessed March 23, 2023).

[B8] CDC Centers for Disease Control Prevention (2022). Health Insurance Portability and Accountability Act of 1996 (HIPAA). Available online at: https://www.cdc.gov/phlp/publications/topic/hipaa.html (accessed December 24, 2022).

[B9] Cheng H. M. Chiou L. J. Chen T. C. Sung S. H. Chen C. H. Lang H. C.. (2019). Real-world cost-effectiveness of drug-eluting stents vs. bare-metal stents for coronary heart disease-A five-year follow-up study. Health Policy 123, 229–234. 10.1016/j.healthpol.11010 30578037

[B10] Davenport T. Kalakota R. (2019). The potential for artificial intelligence in healthcare. Future Healthcare J. 6, 94–98. 10.7861/futurehosp.6-2-94 31363513 PMC6616181

[B11] Eindhoven D. C. Hilt A. D. Zwaan T. C. Schalij M. J. Borleffs C. J. W. (2018b). Age and gender differences in medical adherence after myocardial infarction: women do not receive optimal treatment—The Netherlands claims database. Eur. J. Prev. Cardiol. 25, 181–189. 10.1177/2047487317744363 29164916

[B12] Eindhoven D. C. van Staveren L. N. van Erkelens J. A. Ikkersheim D. E. Cannegieter S. C. Umans V. A. W. M.. (2018a). Nationwide claims data validated for quality assessments in acute myocardial infarction in the Netherlands. J. Neth. Soc. Cardiol. Netherlands Heart Found. 26, 13–20. 10.1007/s12471-017-1055-3 29119544 PMC5758448

[B13] Epping J. Geyer S. Eberhard S. Tetzlaff J. (2021). Völlig unterschiedlich oder doch recht ähnlich? Die soziodemografische Struktur der AOK Niedersachsen im Vergleich zur niedersächsischen und bundesweiten Allgemein- und Erwerbsbevölkerung. Gesundheitswesen Bundesverband der Arzte des Offentlichen Gesundheitsdienstes. 83, S77–86. 10.1055./a-1553-3565 34695865

[B14] Eurostat Statistics Explained (2022). Cardiovascular diseases statistics. Available online at: https://ec.europa.eu/eurostat/statistics-explained/index.php?title=Cardiovascular_diseases_statisticsandoldid=576397

[B15] Federal Institute for Drugs Medical Devices (2022). ILCD to ICD-10. Available online at: https://www.bfarm.de/EN/Code-systems/Classifications/ICD/ICD-10-WHO/History/ilcd-to-icd-10.html (accessed November 14, 2022).

[B16] Gothe H. (2008). Pharmakoepidemiologie. Nutzung der Arzneimittelverordnungsdaten. Bundesgesundheitsblatt Gesundheitsforschung. Gesundheitsschutz 51, 1145–1154. 10.1007/s00103-008-0649-8 18985408

[B17] Hickson R. P. Robinson J. G. Annis I. E. Killeya-Jones L. A. Korhonen M. J. Cole A. L.. (2017). Changes in statin adherence following an acute myocardial infarction among older adults: patient predictors and the association with follow-up with primary care providers and/or cardiologists. J. Am. Heart Assoc. 6,7106. 10.1161./JAHA.117.007106 29051213 PMC5721894

[B18] Hirsch J. A. Leslie-Mazwi T. M. Nicola G. N. Barr R. M. Bello J. A. Donovan W. D.. (2015). Current procedural terminology a primer. J. Neurointervent. Surgery. 7, 309–312. 10.1136/neurintsurg-2014-011156 24589819

[B19] Hirsch J. A. Nicola G. McGinty G. Liu R. W Barr R. M Chittle M. D.. (2016). ICD10: history and context AJNR. Am. J. Neuroradiol. 37, 596–599. 10.3174/ajnr.A4696 26822730 PMC7960170

[B20] Hofmann R. James S. K. Jernberg T. Lindahl B. Erlinge D. Witt N.Centers for Disease Control Prevention. (2017). Oxygen therapy in suspected acute myocardial infarction. N. Engl. J. Med. 377, 1240–1249. 10.1056/NEJMoa1706222 28844200

[B21] Hohnloser S. H. Basic E. Nabauer M. (2019). Changes in oral anticoagulation therapy over one year in 51,000 atrial fibrillation patients at risk for stroke: a practice-derived study. Thromb. Haemost. 119, 882–893. 10.1055/s-0039-1683428 30900220

[B22] Ibrahim S. A. Charlson M. E. Neill D. B. (2020). Big data analytics and the struggle for equity in health care: the promise and perils. Health Equity. 4, 99–101. 10.1089/heq.2019.0112 32258961 PMC7133425

[B23] ICD Codes (2022). The switch from ICD-9 to ICD-10: When and why. Available online at: https://icd.codes/articles/icd9-to-icd10-explained (accessed November 14, 2022).

[B24] Jaunzeme J. Eberhard S. Geyer S. (2013). Wie “repräsentativ” sind GKV-Daten? Demografische und soziale Unterschiede und Ähnlichkeiten zwischen einer, GKV-Versichertenpopulation, der Bevölkerung Niedersachsens sowie der Bundesrepublik am Beispiel der AOK. Niedersachsen. Bundesgesundheitsblatt Gesundheitsforschung Gesundheitsschutz. 56, 447–454. 10.1007/s00103-012-1626-9 23334292

[B25] Jeffrey W. (2022). What Is Data Volume And How To Face Discovery Challenges In Healthcare. Available online at: https://www.jdsupra.com/legalnews/what-is-data-volume-and-how-to-face-1804605/#:~:text=The%20healthcare%20sector%20generates%20more,other%20forms%20of%20healthcare%20data (accessed November 21, 2022).

[B26] Jeschke E. Searle J. Günster C, Baberg, H. T. Dirschedl P. Levenson B.. (2017). Drug-eluting stents in clinical routine: a 1-year follow-up analysis based on German health insurance administrative data from. 2008 to 2014. BMJ Open. 7, e017460. 10.1136/bmjopen-2017-017460 28756388 PMC5642747

[B27] Jones L. K. Pulk R. Gionfriddo M. R. Evans M. A. Parry D. (2018). Utilizing big data to provide better health at lower cost. *Am. J. Health Sys Pharm*. AJHP. 75, 427–435. 10.2146/ajhp170350 29572311

[B28] Kaushik K Bhardwaj A. Dwivedi A. D. Singh R. Machine learning-based regression framework to predict health insurance premiums. Int. J. Environ. Res. Public Health. (2022) 19, 13. 10.3390./ijerph19137898 35805557 PMC9265373

[B29] Kim Y. J. Park H. (2019). Improving prediction of high-cost health care users with medical check-up data. Big Data. 7, 163–175. 10.1089/big.2018.0096 31246499

[B30] Kimm H. Yun J. E. Lee S. H. Jang Y. Jee S. H. (2012). Validity of the diagnosis of acute myocardial infarction in korean national medical health insurance claims data: the korean heart study. Korean Circ. J. (2012) 42, 10–15. 10.4070/kcj.421.10 22363378 PMC3283749

[B31] Kiyota Y. Schneeweiss S. Glynn R. J. Cannuscio C. C. Avorn J. Solomon D. H.. (2004). Accuracy of Medicare claims-based diagnosis of acute myocardial infarction: estimating positive predictive value on the basis of review of hospital records. Am. Heart J. 148, 99–104. 10.1016/j.ahj.02013 15215798

[B32] Koopman C. Vaartjes I. Heintjes E. M. Spiering W. van Dis I. Herings R. M. C.. (2013). Persisting gender differences and attenuating age differences in cardiovascular drug use for prevention and treatment of coronary heart disease, 1998–2010. Eur. Heart J. 34, 3198–3205. 10.1093/eurheartj/eht368 24046432

[B33] Liao C. T. Hsieh T. H. Shih C. Y. Liu P. Y. Wang J. D. (2021). Cost-effectiveness of percutaneous coronary intervention versus medical therapy in patients with acute myocardial infarction: real-world and lifetime-horizon data from Taiwan. Sci. Rep. 11, 5608. 10.1038/s41598-021-84853-y 33692425 PMC7947011

[B34] Makowski L. Köppe J. Engelbertz C. Kühnemund L. Fischer A. J, Lange, S. A.. (2022). Sex-related differences in treatment and outcome of chronic limb-threatening ischaemia: a real-world cohort. Eur. Heart J. 43, 1759–1770. 10.1093/eurheartj/ehac016 35134893 PMC9076397

[B35] Malyar N. Fürstenberg T. Wellmann J. Recent trends in morbidity in-hospital outcomes of in-patients with peripheral arterial disease: a nationwide population-based analysis. Eur. Heart J. (2013) 34, 2706–2714. 10.1093./eurheartj/eht288 23864133

[B36] Mangiapane S. Busse R. (2011). Prescription prevalence and continuing medication use for secondary prevention after myocardial infarction: the reality of care revealed by claims data analysis. Dtsch. Arztebl. Int. 108, 856–862. 10.3238/arztebl.2011.0856 22259640 PMC3258576

[B37] Nestler S. Kreft D. Donndorf P. Ince H. Doblhammer G. (2022). Stents vs. bypass surgery: 3-year mortality risk of patients with coronary interventions aged 50+ in Germany. J. Cardiothorac. Surg. 17, 246. 10.1186./s13019-022-02014-2 36183091 PMC9526318

[B38] Nicholls S. G. Langan S. M. Sørensen H. T. Petersen I. Benchimol E. I. (2016). The RECORD reporting guidelines: meeting the methodological and ethical demands of transparency in research using routinely-collected health data. Clin. Epidemiol. 8, 389–392. 10.2147/CLEP.S110528 27799820 PMC5076545

[B39] Obi E. N. Swindle J. P. Turner S. J. Russo P. A. Altan A. (2017). Healthcare Costs Among patients with heart failure: a. comparison of costs between matched decedent survivo. Cohorts. 34, 261–276. 10.1007./s12325-016-0454-y 27933568

[B40] Pastorino R. De Vito C. Migliara G. Glocker K. Binenbaum I. Ricciardi W. (2019). Benefits and challenges of Big Data in healthcare: an overview of the European initiatives. Eur. J. Public Health 29(Supplement_3), 23–27. 10.1093./eurpub/ckz168 31738444 PMC6859509

[B41] Radzimanowski M. Gallowitz C. Müller-Nordhorn J. Rieckmann N. Tenckhoff B. (2017). Physician specialty and long-term survival after myocardial infarction—A study including all German statutory health insured patients. Int. J. Cardiol. (2018) 251, 1–7. 10.1016/j.ijcard.10048 29092757

[B42] Raghupathi W. Raghupathi V. (2014). Big data analytics in healthcare: promise and potential. Health Inform. Sci. Sys. 2, 3. 10.1186/2047-2501-2-3 25825667 PMC4341817

[B43] Reinecke H. Unrath M. Freisinger E. Bunzemeier H. Meyborg M. Lüders F.. (2015). Peripheral arterial disease and critical limb ischaemia: still poor outcomes and lack of guideline adherence. Eur. Heart J. 36, 932–938. 10.1093/eurheartj/ehv006 25650396

[B44] Riedel O. Ohlmeier C. Enders D. Elsässer A. Vizcaya D. Michel A.. (2018). The contribution of comorbidities to mortality in hospitalized patients with heart failure. J. Germ. Cardiac. Soc. 107, 487–497. 10.1007/s00392-018-1210-x 29404680

[B45] Rumsfeld J. S. Joynt K. E. Maddox T. M. (2016). Big data analytics to improve cardiovascular care: promise and challenges. Nat. Rev. Cardiol. 13, 350–359. 10.1038/nrcardio.2016.42 27009423

[B46] Sabaté M. Vidal X. Ballarin E. Rottenkolber M. Schmiedl S. Grave B.. (2021). Adherence to direct oral anticoagulants in patients with non-valvular atrial fibrillation: a cross-national comparison in six european countries (2008–2015). Front. Pharmacol. 12, 682890. 10.3389/fphar.2021.682890 34803665 PMC8596153

[B47] Sawicki O. A. Mueller A. Glushan A. Breitkreuz T. Wicke F. S. Karimova K.. (2020). Intensified ambulatory cardiology care: effects on mortality and hospitalisation-a comparative observational study. Sci. Rep. 10, 14695. 10.1038/s41598-020-71770-9 32895445 PMC7477232

[B48] Shafazand S. Yang Y. Amore E. O'Neal W. Brixner D. A. (2010). retrospective, observational cohort analysis of a nationwide database to compare heart failure prescriptions and related health care utilization before and after publication of updated treatment guidelines in the United States. Clin. Ther. (2010) 32, 1642–1650. 10.1016/j.clinthera.08002 20974322

[B49] Statistisches Bundesamt (2022). Cost of illness by diseases and gender in Euro per inhabitant. Available online at: https://www.destatis.de/EN/Themes/Society-Environment/Health/Cost-Illness/Tables/disease-categories-gender.html;jsessionid=F175E4714052E9BC8B5F28623FC78466.live711

[B50] Swart E. Bitzer E. M. Gothe H. Harling M. Hoffmann F. Horenkamp-Sonntag D.. (2016). STandardisierte BerichtsROutine für Sekundärdaten Analysen (STROSA) - ein konsentierter Berichtsstandard für Deutschland, Version 2. Gesundheitswesen (Bundesverband der Arzte des Offentlichen Gesundheitsdienstes). 78, e161. 10.1055./s-0042-112008 27428525

[B51] Swart E. Gothe H. Geyer S. Jaunzeme J. Maier B. Grobe T. G.. (2015). Gute Praxis Sekundärdatenanalyse (GPS): Leitlinien und Empfehlungen. Gesundheitswesen (Bundesverband der Arzte des Offentlichen Gesundheitsdienstes). 77, 120–126. 10.1055/s-0034-1396815 25622207

[B52] Swart E. Gothe H. Hoffmann F. Ihle P. Semler S. C. March S.. (2021). Jetzt die Weichen stellen für ein leistungsfähiges Forschungsdatenzentrum Gesundheit. Gesundheitswesen. Bundesverband der Arzte des Offentlichen Gesundheitsdienstes. 83, S139–S141) 10.1055./a-1537-9722 34695868

[B53] Thesmar D. Sraer D. Pinheiro L. Dadson N. Veliche R. Greenberg P.. (2019). Combining the power of artificial intelligence with the richness of healthcare claims data: opportunities and challenges. PharmacoEconomics 37, 745–752. 10.1007/s40273-019-00777-6 30848452

[B54] Ulrich R. Pischon T. Robra B-. P. Freier C. Heintze C. Herrmann W. J.. (2019). Health care utilisation and medication one year after myocardial infarction in Germany—A claims data analysis. Int. J. Cardiol. (2020). 300, 20–26. 10.1016/j.ijcard.07050 31371116

[B55] van Brabandt H. Neyt M. Devos C. (2013). Effectiveness of catheter ablation of atrial fibrillation in Belgian practice: a cohort analysis on administrative data. Eur. Soc. Cardiol. 15, 663–668. 10.1093/europace/eut004 23388182

[B56] van der Galiën O. P. Hoekstra R. C. Gürgöze M. T. Manintveld O. C. van den Bunt M. R. Veenman C. J.. (2021). Prediction of long-term hospitalisation and all-cause mortality in patients with chronic heart failure on Dutch claims data: a machine learning approach. *BMC Med*. Inform. Decis. Mak. 21, 303. 10.1186/s12911-021-01657-w 34724933 PMC8561992

[B57] Wennberg J. E. (2010). Tracking Medicine: A Researcher's Quest to Understand Health Care. New York: Oxford University Press.

[B58] WHO Collaborating Centre for Drug Statistics Methodology (2022). International language for drug utilization research. Available online at: https://www.whocc.no/ (accessed November 14, 2022).

[B59] Wilson J. F. (2006). Health insurance portability and accountability act privacy rule causes ongoing concerns among clinicians and researchers. Ann. Intern. Med. 145, 313–316. 10.7326/0003-4819-145-4-200608150-00019 16908928

[B60] Wolf M. S. Bennett C. L. (2006). Local perspective of the impact of the HIPAA privacy rule on research. Cancer 106, 474–479. 10.1002/cncr.21599 16342254

[B61] World Health Organization (2022). The top 10 causes of death. Available online at: https://www.who.int/news-room/fact-sheets/detail/the-top-10-causes-of-death (accessed December 22, 2022).

[B62] Zou J. Schiebinger L. A. I. (2018). Can be sexist and racist—it's time to make it fair. Nature 559, 324–326. 10.1038/d41586-018-05707-8 30018439

